# The Tagmatized Echinoderm

**DOI:** 10.1093/iob/obag040

**Published:** 2026-07-15

**Authors:** R L Turner

**Affiliations:** Department of Ocean Engineering and Marine Sciences, Florida Institute of Technology, Melbourne 32901 FL, USA

## Abstract

Tagmosis is a derived condition of metamerism in annelids, arthropods, and chordates in which groups of adjacent segments are modified to perform specific functions. Examples are the clitellum of earthworms, the carapace of lobsters, and the sacrum of tetrapods, to mention a few. The argument is advanced here that examples of tagmosis are not hard to find among echinoderms, a fourth major group of metameric animals; but the degree of tagmosis is weak in most cases. The first few segments of the axial skeleton are often conjoined as a perioral tagma (e.g., the jaws and other parts of the oral frame of asteroids and ophiuroids and the lantern supports of echinoids). Several segments beyond the oral frame unite the disc with the base of the arm in ophiuroids. Crinoid pinnules occur in groups along the arms as oral, genital, and feeding pinnules. Porcellanasterid seastars have cribriform organs along the marginal ossicles on the base of the arm for generation of respiratory currents. Well-developed podial pores and podia are restricted to basal segments of the ophiuroid genus *Ophiomusium*. Ambulacral plates of most post-Paleozoic echinoids occur in clusters that result in arcs of variable numbers of pore pairs and spine tubercles, giving rise to several non-cidaroid tagmatized patterns. The strongest degree of tagmosis occurs in the bilaterally symmetrical echinoids, with petals, phyllodes, plastrons, and frontal ambulacra; and tagmosis differs among the axes. Tagmosis is absent in extant holothuroids, bodies of which are almost entirely composed of extra-axial elements. Tagmosis occurs in the oral regions of many extinct echinoderm classes, and the ambulacra are tagmatized in some, particularly in blastozoan classes and those with branched ambulacra.

## German

Tagmose ist eine abgeleiteter Zustand der Metamerie bei Ringelwürmern, Gliedertiere, und Chordaten, bei der Gruppen benachbarter Segmente so modifiziert werden, dass sie spezifische Funktionen erfüllen. Beispiele dafür sind das Clitellum von Regenwürmern, der Carapax von Hummern, und das Kreuzbein von Tetrapoden, um nur einige zu nennen. Hier wird der Beweisgrund vorgebracht, dass Beispiele für Tagmose bei den Stachelhäutern—einer vierten Hauptgruppe metamerer Tiere—nicht schwer zu finden sind; jedoch ist der Grad der Tagmose in den meisten Fällen gering. Die ersten wenigen Segmente des Achsenskeletts sind oft zu einem perioralen Tagma verbunden (z.B., die Kiefer und andere Teile des Mundapparats von Seesternen und Schlangensternen sowie die Laternenstützen von Seeigeln). Bei Schlangensternen verbinden mehrere Segmente jenseits des Mundapparats die Scheibe mit der Armbasis. Die Fiederblättchen der Seelilien (Crinoidea) treten gruppenweise entlang der Arme als orale, genitale, und Nahrungspinnulae auf. Porcellanasteriden besitzen an den Randossikeln der Armbasis kribriforme Organe zur Erzeugung von Atemströmen. Gut entwickelte Podialporen und Podien sind auf die basalen Segmente der Schlangensterngattung *Ophiomusium* beschränkt. Die Ambulakralplatten der meisten post-paläozoischen Seeigel treten in Clustern auf, die Bögen mit unterschiedlicher Anzahl an Porenpaaren und Stachelhöckern bilden und so verschiedene, nicht-cidaroide tagmatisierte Muster entstehen. Die stärkste Ausprägung der Tagmose findet sich bei den bilateral symmetrischen Seeigeln mit ihren Petalen, Phyllodien, Plastronen, und Frontalambulacren; die Tagmose unterscheidet sich zwischen den Achsen. Rezente Seegurken, deren Körper fast vollständig aus extraaxialen Elementen bestehen, weisen keine Tagmose auf. Tagmose kommt in den Mundregionen vieler ausgestorbener Stachelhäuterklassen vor, und die Ambulakren sind bei einigen, insbesondere bei Blastozoen und solchem mit verzweigten Ambulakren, tagmatisiert.

## Introduction

The organization of the human axial skeleton is familiar to many people, especially to the elderly. In addition to cranial afflictions, some patients have had fusion of cervical vertebrae, others correction of scoliosis of thoracic vertebrae, others laminectomy for stenosis of lumbar vertebrae, others sacroplasty for a fractured sacrum, and yet others massage therapy for coccydynia (tailbone pain). Each section of the vertebral column is a tagma (pl. tagmata; Greek τάγμα, “an ordering or arrangement of things”), a morphologically and functionally specialized group of segments. The segmented (metameric) bodies of many vertebrate animals are strongly tagmatized: the neck of giraffes, the shell of turtles, the trunk of porpoises, and the tail of stingrays are distinctly different in morphology and function from other tagmata of their axial skeleton. Strong axial specialization (tagmosis) is the norm except for some groups, such as snakes, in which the degree of tagmosis is reduced from the body plan of their Mesozoic ancestors (Hsiang et al. 2015; Kardong 2019).

The type of segmentation exhibited by vertebrates is metamerism (Clark 1964), although some authorities use “segmentation” as a synonym for “metamerism” (Willmer 1990); terminology continues to be unsettled (Minelli 2017). Metamerism in animals is a serial (linear) repetition of parts (modules, segments, metameres) of mesodermal origin, appearing subterminally early in development; authors distinguish this form of segmentation and serial repetition from others, as in tapeworms and some mollusks (Clark 1964; Russell-Hunter 1979; Willmer 1990). Fusco and Minelli (2013) report that tagmosis occurs in the bodies of “many bilaterian taxa,” and they explain that “tagmosis represents a form of higher modularity along the main body axis” of metameric animals.

Two other groups that classically are viewed as metameric are arthropods and the annelid worms (Clark 1964; Russell-Hunter 1979; Willmer 1990). Members of these groups also typically express tagmosis. Familiar cases among arthropods are the carapace and abdomen of lobsters; the cephalon, trunk, and pygidium of trilobites; and the head, thorax, and abdomen of insects. Tagmosis is typically expressed in arthropods, but centipedes are an example of arthropods with minimal tagmatization. Tagmata of annelids are weakly expressed in leeches, earthworms, and many marine bristleworms. Some bristleworms, by contrast, are strongly tagmatized as in the lugworms and parchment tube worms. Willmer (1990) stated that “tagmosis with functional specialization of particular sections is a predictable consequence of segmentation”; that is, tagmosis often is a convergent feature among metameric animals (Moore and Willmer 1997).

For more than a century, the prevailing view of the echinoderm body plan has been one of oligomeric organization (with two or three regions that are not serial repetitions); but echinoderms have recently been argued to express metamerism along each of their several body axes (Turner 2024); this view of metamerism in echinoderms has already received some attention in the echinoderm literature (Isaeva 2024, 2025; Formery et al. 2025; Rozhnov and Tkacheva 2025). Turner’s (2024) arguments drew heavily from another long-known phenomenon of subapical addition of segments, formally named the Ocular Plate Rule (OPR) by Mooi and David (1993), and a more recently described feature of the echinoderm body plan called the Extraxial-Axial Theory (EAT; Mooi et al. 1994). The OPR describes the addition of segments (metameres) behind the ocular plate at the tip of a growing ambulacrum (the axial skeleton) of echinoderms. The EAT distinguishes between those parts of the echinoderm body derived from the axial skeleton of the juvenile and those parts (extraxial) derived from the larval body. Metamerism, the OPR, and the EAT coincide well with each other (Turner 2024). If Willmer’s (1990) observation is correct that metameric animals often express tagmosis as a derived condition, tagmosis might also be expressed in echinoderms. The purposes of this study were to examine the degrees to which and the ways in which morphological and functional specialization occurs along the metameric axes of echinoderms and to argue that these are examples of tagmosis. Emphasis is placed on the five extant classes: Crinoidea, Asteroidea, Ophiuroidea, Echinoidea, and Holothuroidea.

## Methods

Examples of tagmosis were extracted from general literature on echinoderms and from monographs on the five extant and some of the more than two dozen extinct classes (Deline et al. 2020). In addition to literature cited in sections that follow, the works of Bather (1900), Cuénot (1948), Hyman (1955), and Byrne and O’Hara (2017) were consulted because of their extensive inclusion of morphology of all extant classes. The focus was on the axial skeleton and some associated extraxial structures based on the EAT (Mooi et al. 1994, 2005; Mooi and David 1997, 2000; David et al. 2000).

### The perioral tagma

In most extant and extinct echinoderm classes, the axial skeleton grows according to the OPR distally from the oral (proximal) region of the juvenile. The most proximal (oldest) elements (ossicles) of the axial skeleton have been identified in all classes (Deline et al. 2020; Woodgate et al. 2025). Along with some extraxial elements, the proximal ossicles form a perioral skeleton, which can be viewed as a tagma in most echinoderms, even if the rest of each axis (the ambulacrum) is not further tagmatized. The perioral tagma is specialized to support feeding activities and to bind together the several ambulacra.

**Fig. 1:**
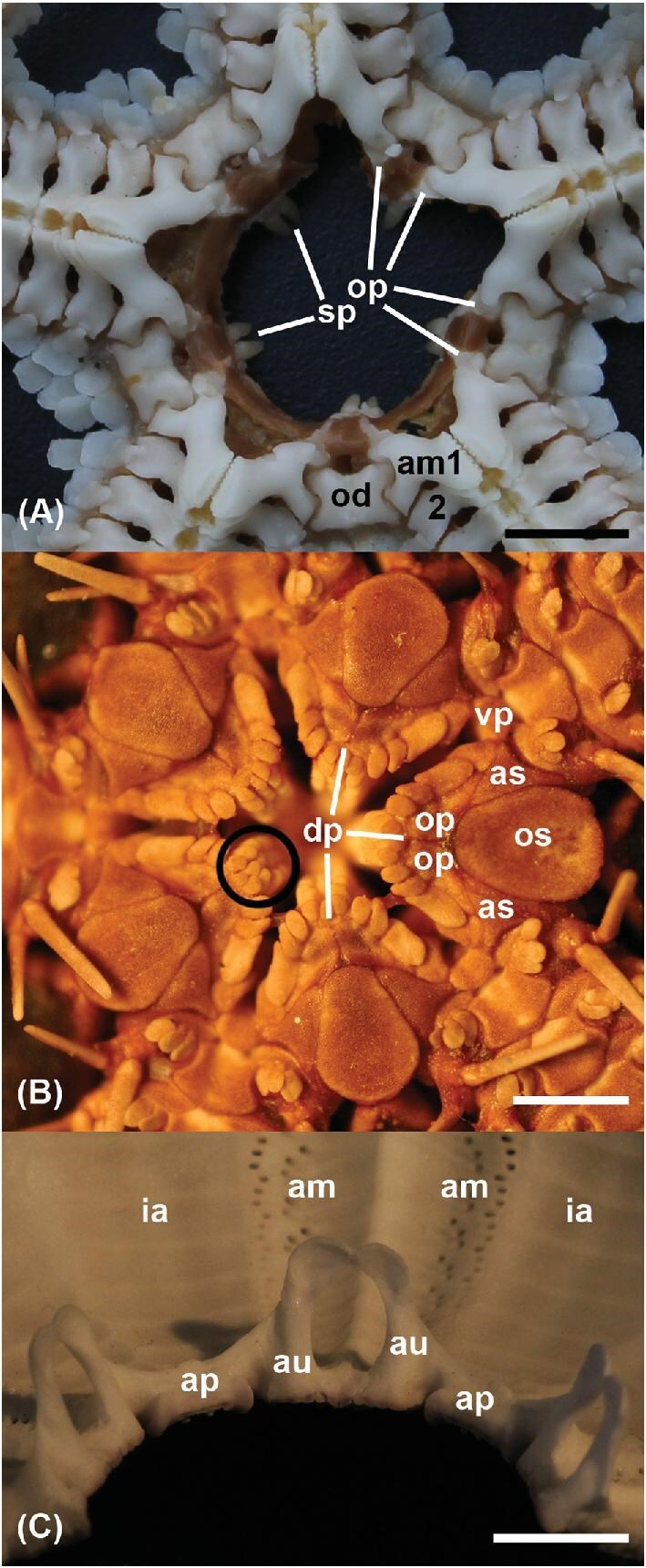
Examples of perioral tagmata in echinoderms. (A) The oral frame of *Ctenodiscus crispatus* (Asteroidea; dissected and briefly treated with bleach, aboral view) includes 35 elements: 10 first ambulacral ossicles (am1), each representing the conjoined first and second ambulacral ossicles in the juvenile rudiment; 10 second ambulacral ossicles (2); 10 oral plates (op), mostly hidden from view by the ambulacral ossicles but revealed by some of their oral spines (sp); 5 odontophores (od), unpaired interradial extraxial elements present in many families of seastar. Scale bar 5 mm. (B) The oral frame of *Ophiomastix wendtii* (Ophiuroidea; view of oral surface) includes 30 externally visible ossicles as well as other internal ones: 5 oral shields (os); 10 adoral shields (as); 10 oral plates (op); 5 first ventral arm plates (vp); 5 dental plates (dp, position indicated but mostly obscured by clusters of their tooth papillae [encircled]). Scale bar 2 mm. (C) The perignathic girdle of *Lytechinus variegatus* (Echinoidea, dissected, treated with bleach, internal view of test) consists of 5 pairs of auricles (au), each pair at the proximal end of an ambulacral column (am), and 5 apophyses (ap) of the interambulacral columns (ia). Scale bar 5 mm.

**Fig. 2:**
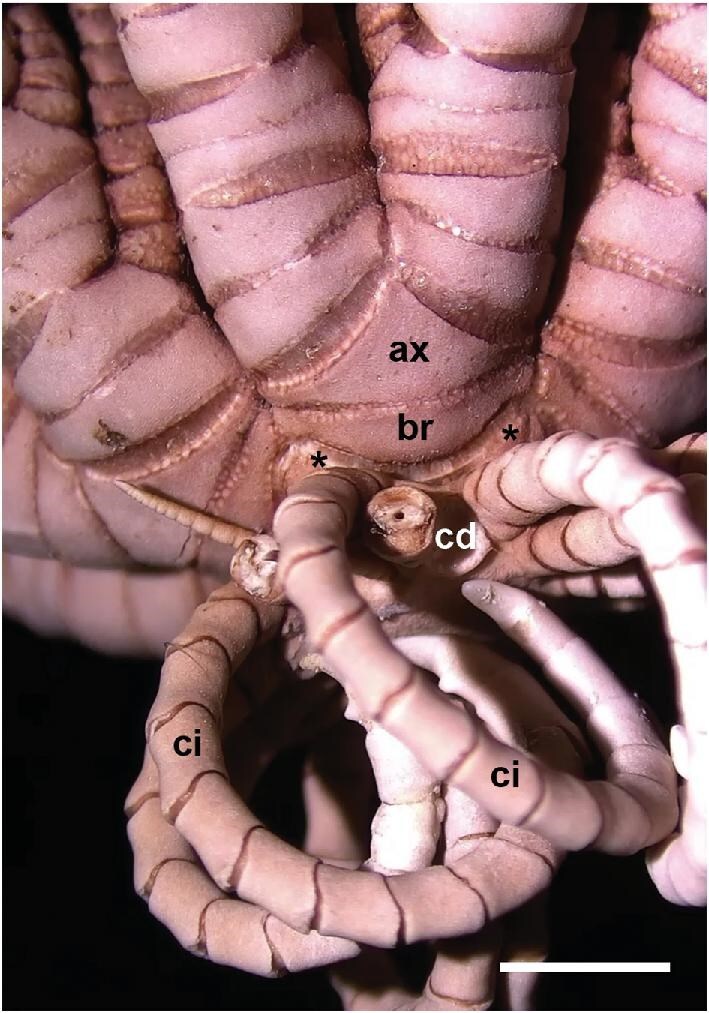
*Comactinia meridionalis* tagmata. The dorsal-most part of the body of this 10-armed featherstar consists of a tight cluster of ossicles: a centrodorsal (cd, mostly hidden by cirri), to which are attached a circlet of cirri (ci) and a ring of five basals (hidden). Five arm bases arise from a second ring of five radials, one partly showing between the asterisks (*). Radials give rise to the first tagma of the axial skeleton: one brachial (br) and an axillary (ax). Two branches, free arms, arise from the axillary, each branch a long series of brachials that form a second (distal) tagma. Scale bar 2 mm.

If the calcareous ring of extant holothuroids is axial (but see Mooi and David 1997), it would be the only part of the axial skeleton and, therefore, not technically a tagma for lack of distal elements (Mooi and David 1997). Indeed, some elasipod holothuroids lack ossicles, including the calcareous ring (Rowe et al. 2017; Gebruk & Kremenetskaia 2025). On the other hand, extinct holothuroids such as *Palaeocucumaria* and the holothuroid sister group the Ophiocistioidea bear short ambulacra with tentacles clustered near the perioral tagma (Smith and Reich 2013). The axes of asteroids and ophiuroids are bound by the oral frame (Fig. 1A, B), which consists of the modified first few elements of the axial skeleton along with some extraxial ossicles (Hyman 1955; Fell 1963; Mooi and David 2000; Hendler 2018). The perignathic girdle of echinoids (Fig. 1C) strengthens the oral end of the globose test and serves for attachment of muscles of Aristotle’s lantern in those families that have a lantern (Hyman 1955; Kier 1970); the peristomial membrane between the perignathic girdle and the mouth varies in composition among echinoid taxa (David et al. 1995) and might or might not be viewed as a more adoral tagma. The calyx (central body) of extant crinoids is formed by the sets of five basal and radial ossicles (extraxial) and usually one or more basal elements of each arm (fixed brachials, axial; Ausich et al. 1999), from which the free arms extend (Fig. 2). Whether the oral elements are homologous (Kammer et al. 2013) or not (Guensburg et al. 2016), the oral frames of the several extinct blastozoan classes, the extinct edrioasteroids, and Paleozoic crinoids form tagmata that are distinct morphologically from the distal components (ambulacra) of their axial skeletons. Tagmatization of the ambulacra is described in the following sections.

### Ambulacral tagmatization in extinct classes

The axial skeleton distal to the oral tagma has been recognized as ambulacral (podia bearing) in all extinct classes of the echinoderms (David et al. 2000; Deline et al. 2020; Rahman and Zamora 2024; Woodgate et al. 2025). In some members of classes, the ambulacra are further tagmatized, most notably in those with branching ambulacra. In class Blastoidea, there are species with brachioles (feeding appendages; Sprinkle 1973) arising from the proximal and distal regions of the ambulacra differing in morphology, configuration, and presumed function (Waters et al. 2024). In members of some blastozoan classes, brachioles arise periodically from the ambulacra, forming an apparent branching pattern (David et al. 2000) in which the ambulacral segments beyond a brachiole to the branch point bearing the next brachiole would be a tagma as in branching species of extant echinoderm classes (see below). In other blastozoans, the ambulacra may be branched (Gil Cid et al. 2004; Guensburg et al. 2010; Nardin et al. 2010). In the 2-1-2 pattern of ambulacral symmetry demonstrated in a number of extinct classes (e.g., class Edrioasteroidea but including various blastozoan classes), two of the three ambulacra that arise from the perioral tagma branch (Sumrall and Wray 2007; Briggs et al. 2017). The base of each lateral ambulacrum would be a proximal ambulacral tagma and each branch would be a distal ambulacral tagma. Stylophorans, a non-blastozoan class, have an asymmetric or symmetric theca (the body, which is extraxial) from which one biserial aulacophore (feeding and locomotory “appendage”) extends anteriorly (Lefebvre 2003). Although the aulacophore appears to consist of two tagmata separated by a mouth, the proximal part is extraxial; the only axial component is the feeding and locomotory arm (distal aulacophore), an ambulacrum that is not further specialized (David et al. 2000; Lefebvre 2003; Lefebvre et al. 2006, 2019).

### Crinoidea

Almost all extant crinoids have branched arms. Arms of most species branch once to produce 10 arms from the original 5 arms (Fig. 2), but arms of many species branch more frequently than once, up to 9 times in some species (Clark 1921; Hyman 1955; Messing and Dearborn 1990; Ausich et al. 1999). Each section (brachitaxis or division series) consists of a proximal ossicle (brachial), a series of intermediate brachials of variable number, and the most distal brachial. If the distalmost brachial is a branch point, it is called an axillary (Fig. 2). Each brachitaxis is a tagma and might consist of different kinds of brachials depending on the type of joint by which adjacent brachials articulate. In 10-armed crinoids, arms have two tagmata: a basal tagma of one brachial beyond the radial, an axillary, and perhaps one or more brachials between them; a distal tagma of the remainder of the brachials (Fig. 2). The arms of 5-armed crinoids are not tagmatized with regard to brachials aside from the contribution of basal ones to the structure of the calyx.

**Fig. 3:**
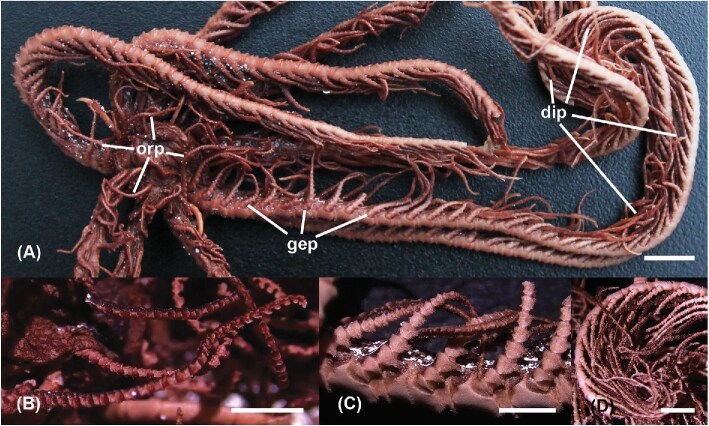
*Comactinia meridionalis* and pinnules. (A) Whole preserved specimen with most of its 10 arms visible and with examples of oral (orp), genital (gep), and distal pinnules (dip) indicated by pointers. Scale bar 5 mm. (B) An oral pinnule in foreground, with others in background. Scale bar 2 mm. (C) Five genital pinnules of a non-gravid specimen. Scale bar 2 mm. (D) A distal section of arm with numerous distal (feeding) pinnules. Scale bar 2 mm.

The morphology and arrangement of pinnules on crinoid arms might impose a second pattern of tagmosis (Clark 1921; Hyman 1955; Messing and Dearborn 1990). Pinnules of many crinoids are grouped into oral, genital, and distal pinnules (Fig. 3), each kind of pinnule with distinctive morphology and function. Oral pinnules (Fig. 3B) of two to several basal brachials are tactile, often lack podia, are long, and overlie the tegmen, protecting the soft tissue; they can be morphologically quite different from pinnules on more distal brachitaxes. Genital pinnules (Fig. 3C) bear the gonads, might be the shortest pinnules but increasing in length on distal brachials, and they occur on many brachials in the basal to middle parts of the arm; genital pinnules can lack ambulacral grooves and podia. Distal pinnules (Fig. 3D) are borne on the outer half or more of the arm and are the primary feeding pinnules. Distal pinnules have ambulacral grooves and the three-lobed podia typical of crinoids.

### Asteroidea

**Fig. 4:**
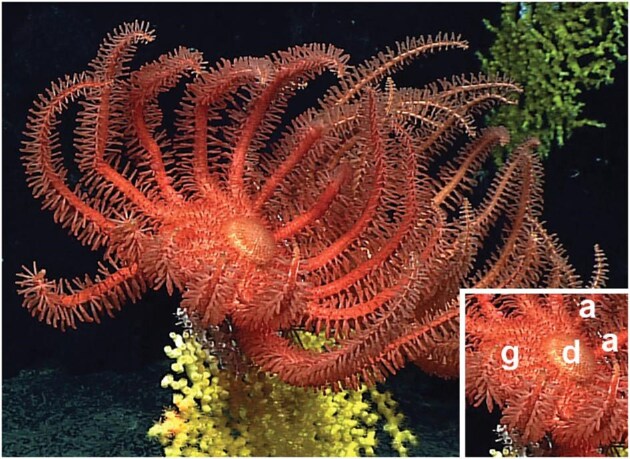
*Novodinia pacifica*, a brisingid seastar with arms clearly demarcated from the small central disc (d, inset). Gonads are not serially arranged in this genus, but the base of each free arm (a) is swollen with the paired gonads, producing a distinct tagma (g). (NOAA Digital Collections, Office of Ocean Exploration and Research, 2015 Hohonu Moana, NOAA Ship *Okeanos Explorer*)

Seastars have a variable distribution of granules and spines along the arms (Clark and Downey 1992), but they rarely demonstrate tagmosis beyond the oral frame (the perioral tagma). Axial elements of ambulacral ossicles and, arguably (Mooi and David 2000), adambulacral and marginal ossicles generally are progressively smaller (and younger) from proximal to distal positions. Beyond the oral frame, basal ossicles of these series and extraxial ossicles intercalated between the series might be considered to be part of the disc; but the demarcation between disc and arm is rarely clear: it is sharp in species of the order Brisingida (Fig. 4) and indistinguishable in a number of species in the order Valvatida that are pentagonal (Clark and Downey 1992). One might argue that brisingid seastars have three tagmata along the axes: oral frame, support of the ventral disc, free arms (Clark and Downey 1992).

**Fig. 5:**
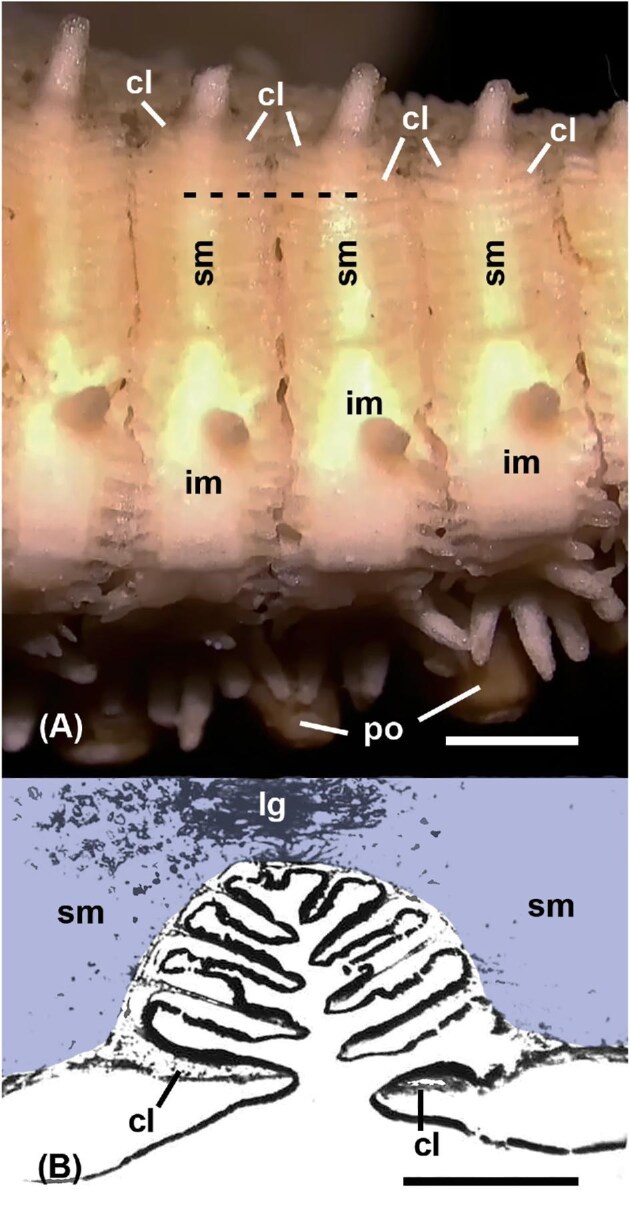
*Ctenodiscus crispatus* cribriform organs. Unlike the restricted condition in porcellanasterid seastars, cribriform organs in this species occur throughout the arm. (A) Four sets of superomarginal (sm) and inferomarginal (im) plates have narrow frontal edges bordered by cover lamellae (cl), consisting of webbed spinules. Podia (po) are visible in this side-view of an arm. Scale bar 1 mm. (B) In this cross-section of two decalcified superomarginal plates (sm, highlighted in blue), several internal lamellae of ciliated spinules form the cribriform organ beneath the cover lamellae (cl). A ligament (lg) joins the superomarginal plates. Scale bar 0.5 mm.

Seastars of the family Porcellanasteridae are exceptional: they bear specialized, marginal, cribriform organs (Madsen 1961). A cribriform organ (Fig. 5) consists of several parallel, vertical, ciliated lamellae between adjacent pairs of supero- and inferomarginal ossicles. Cribriform organs probably serve these infaunal seastars for gas exchange, as demonstrated in a non-porcellanasterid member of the order Paxillosida *Ctenodiscus crispatus* (Shick et al. 1981). The number of cribriform organs from the base of an arm toward the tip in porcellanasterids varies by species from 1 to 16 on each side of the arm (Clark and Downey 1992). One cribriform organ is located at the mid-interradial suture between the marginal pairs of adjacent arms. Additional cribriform organs are confined to the interradial arcs along the margin of the disc in almost all species. They occur between all marginal pairs to the terminal (ocular) plate at the arm tip in only one species of porcellanasterid (Madsen 1961). Cribriform organs are confined to the two series of marginal ossicles and do not influence the adambulacral and ambulacral series in form or function.

Another exception is found among the order Forcipulatida in the arrangement of their podia and gonads (Clark and Downey 1992). As in all asteroids, one segmental pair of podia occurs between adjacent pairs of ambulacral ossicles to each side of the midline, but the position of the podia alternates from near the midline to a distance away from it in some groups. The result is the appearance of four rows of podia, a quadriserial condition, rather than the typical two rows (biserial). In many members of several forcipulatid families, the podia are quadriserial proximally and biserial distally. Gonads are serially arranged with segmental gonopores at least basally along the arms of several genera of family Brisingidae; in *Brisinga synaptoma*, the genital region comprises 60% of the arm length (Fisher 1928; as “*Craterobrisinga synaptoma*”), the distal part of the arm narrowing significantly. Even in species with one pair of gonads per ray, a basal, swollen, genital region of the arm contains the gonads as well as the pyloric caeca (Fig. 4). The swollen genital region can lack the transverse ridges and bands of pedicellariae that characterize the arms of this family.

### Ophiuroidea

It is not encouraging in searching for morphological diversity in brittlestars and their allies to read in Hyman’s (1955) account that “they all look alike.” Many characters change gradually in size, shape, and number along the arm from base to tip without conferring tagmosis on its long series of segments. But tagmosis does occur. Ophiuroids in general have at least three tagmata: axial and extraxial components of the oral frame; basal segments structurally integrated with the disc; the arm free of the disc.

**Fig. 6:**
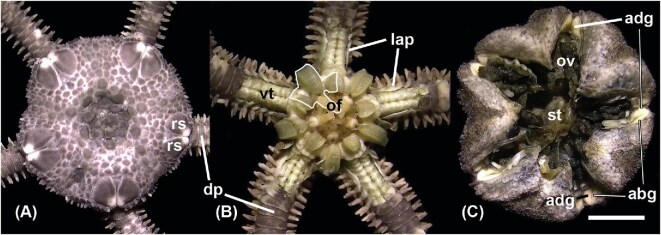
*Ophiophragmus filograneus*, disc attachment. Scale bar 2 mm. (A) Whole specimen. The axial position of the dorsal integument of the disc is occupied by a pair of radial shields (rs). (B) Removal of the dorsal integument reveals the oral frame (of) of the perioral tagma (one of five jaws and its dorsal-most tooth outlined in white) and the scars on the next distal tagma. The vertebrae (vt), which form the “backbone” of the arms, are exposed by the absence of dorsal arm plates (dp) in this tagma. (C) The inverted dorsal integument encloses the stomach (st) and ovaries (ov) in this female specimen. A pair of ossicles, the adradial genital scale (adg) and abradial genital scale (abg), attaches each radial shield to the vertebrae and the spine-bearing lateral arm plates (lap) of each segment in the scar region.

**Fig. 7:**
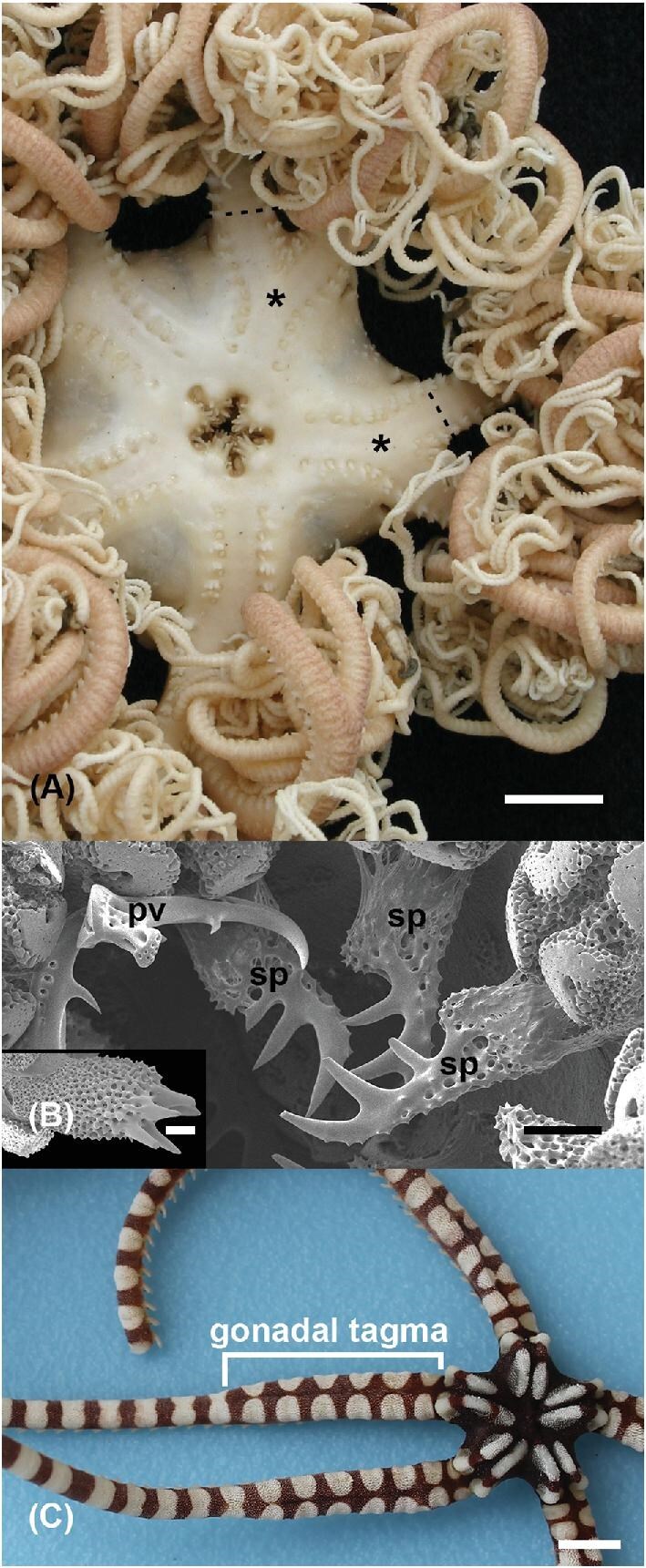
Specialized tagmata in euryalid ophiuroids. (A) The basketstar *Gorgonocephalus eucnemis*, with basal arm branches strengthening the disc. Asterisks (*) show approximate locations of the axillary ossicles (branch point as in crinoids); dashed lines indicate the junctions between the fixed-arm tagma and free-arm tagma. Scale bar 1 cm. (B) *Asteroporpa annulata*, strongly curled arm tip with hooked arm spines (sp), which differ from pedicellarial valves (pv) and fully grown arm spines (inset). Scale bars 100 µm. (C) *Asteroschema tenue*, a snakestar with gonads that extend into the coelom of basal arm segments. Scale bar 5 mm.

In addition to the oral frame (Hendler 2018) (Fig. 1B), a variable number of segments are specialized to secure the overlying disc (Fig. 6). These basal segments lack dorsal arm plates, a condition that produces a dorsal scar if the disc is autotomized or artificially removed; the lateral arm plates often have fewer arm spines than those on more distal segments; the vertebrae are proximo-distally compressed; and the adradial genital plate (genital bar, genital plate) and abradial genital plate (genital scale) (Stöhr 2024) secure the disc to the base of the arm and border the genital slit. The condition in *Astrophiura* (Hyman 1955; Pawson 2018) is an extreme case of the demarcation between basal segments incorporated into the disc and the morphology of the short free arm. The free arm segments of *Astrophiura* lack podia and dorsal and ventral arm plates, whereas segments associated with the disc have large podia and all arm plates, which are an integral part of the disc. Among many genera in the order Euryalida with branching arms, the first branch occurs beneath the disc and further strengthens the central body (Fig. 7A).

In addition to *Astrophiura*, other genera have free arms in which podial pores are lacking or highly reduced (*Aspidophiura, Ophientrema, Ophiolipus, Ophiomusium, Ophioplinthus* [which includes the synonyms *Homalophiura* and *Ophiurolepis*], *Ophiosphalma, Ophiosteira, Ophiuroglypha*; Fell 1960) or in which ventral arm plates are lacking or fragmented (*Ophioholcus, Ophiomoeris* as “*Ophiogyptis*”; Fell 1960). The free arm might be further tagmatized by the nature of the spines, although the demarcation between tagmata might not be sharp: fan-like webbing between arm spines on basal segments in *Ophiopteron* and juvenile *Ophiothrix* for an unconfirmed role in swimming (Ludwig 1888; Mortensen 1932); hooked arm spines on distal segments of some *Ophiolepis* spp. perhaps for feeding (Hendler and Turner 1987) and of *Nannophiura, Ophiodaphne* (as “*Amphilycus*”), *Ophiopholis, Ophiosphaera*, and *Ophiuroglypha*, some of which genera are epizoic (Fell 1960) and might use distal hooked arm spines to cling to the host. Hooked arm spines occur on distal segments of many euryalid basketstars and snakestars; the hooked arm spines differ from the arm spines and pedicellarial valves that occur on most arm segments (Fig. 7B). In *Ophiohelus* and *Ophiotholia*, distal arm segments bear “parasol spines” or umbrella-shaped “pedicellariae,” in addition to typical arm spines, embedded in fleshy sacs (Lyman 1880). Within the arm coelom, the basal two-thirds of the arm in *Ophiocanops fugiens* has metamerically arranged gonads, one pair with gonopores per segment, and an unsegmented extension of the stomach (Mortensen 1932). Unsegmented paired gonads also extend into the base of arms in several genera, including *Asteroschema* (Fig. 7C), *Ophiocreas*, and *Trichaster*, perhaps to accommodate seasonally enlarged gonads or filling of the stomach in species with small discs (Lyman 1882; Fedotov 1926; Mortensen 1932; Hyman 1955; Fell 1960; Hendler 1991).

Arms of many genera in the order Euryalida branch, usually more than once (Lyman 1877; Turner and O’Neill 2023), some basal brachitaxes strengthening the disc, as mentioned above. As in crinoids, each brachitaxis is a tagma, often with a relatively fixed number of segments (Döderlein 1911, 1927, 1930), terminating in an axillary ossicle (Fig. 7A) unless it is the distalmost brachitaxis on the arm. In addition, the morphology and number of arm spines and pedicellariae on the free arm can vary by tagma along the axes. Some basketstars lack arm spines and bands of pedicellariae on basal brachitaxes (Turner and Boucher 2010; Turner et al. 2021), leaving the more distal brachitaxes for grasping prey or substrata.

### Echinoidea

**Fig. 8:**
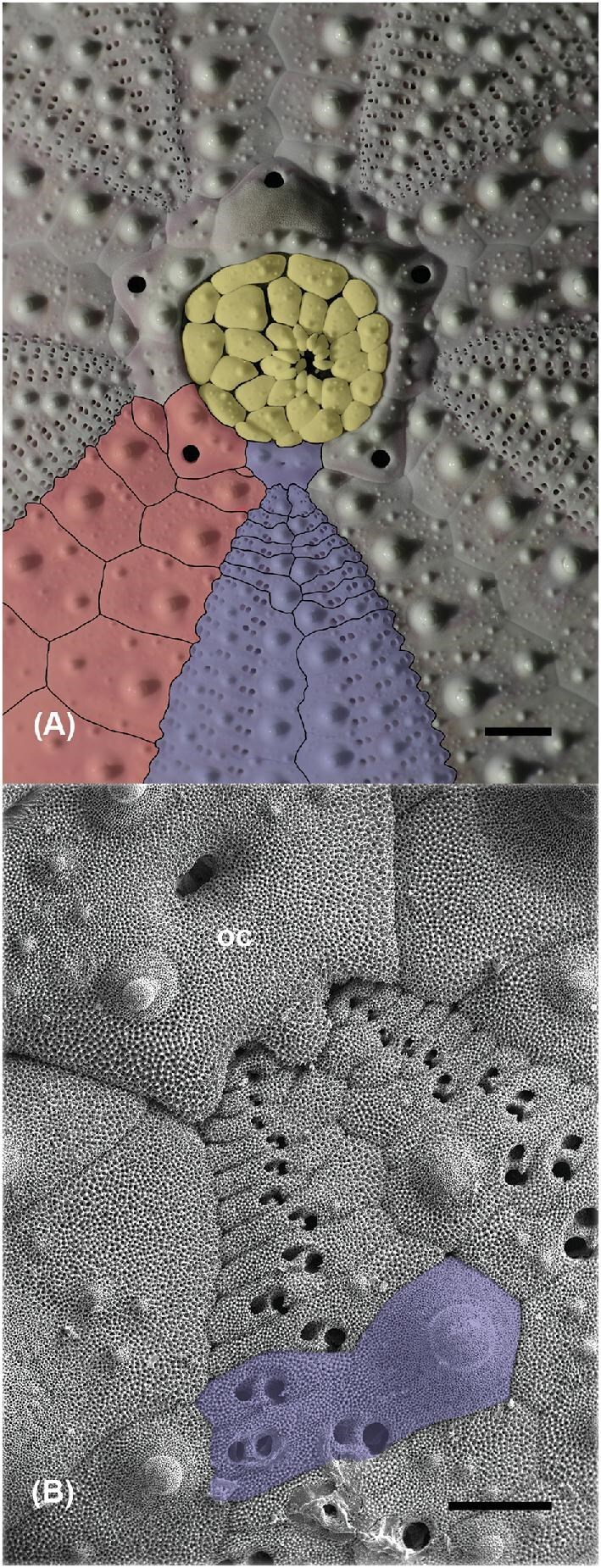
Compound plates of the echinoid *Tripneustes ventricosus*. (A) The globose test is composed of an apical extraxial region (the periproct, or region around the anus; yellow) and twenty columns of axial plates that extend from the periproct to the peristome (region around the mouth). The axial columns are grouped into five double columns of ambulacral plates (blue) and five double columns of interambulacral plates (red). Sutures between some simple and compound plates are drawn here in black. Scale bar 2 mm. (B) New axial plates are produced in the growth region near the periproct, proximal to the ocular plate (oc). Ambulacral ossicles begin as individual units, each with a pore pair and separated from adjacent ambulacral ossicles by sutures. With growth, clusters of three ambulacral plates become conjoined as a compound plate (example in blue), with three pore pairs, one shared tubercle, and obscured sutures. Scale bar 0.5 mm.

Except for the periproct at the apex of the globose test of most echinoids (Fig. 8A), the test is composed entirely of axial elements; Aristotle’s lantern within the test is extraxial (Mooi and David 1997). The test of extant echinoids consists of 20 columns of ambulacral and interambulacral ossicles in two-column clusters (Hyman 1955; Smith 1984). Ambulacral and interambulacral columns rarely have the same number of ossicles (Fig. 8A). An ambulacral ossicle bears a pair of pores, each pair associated with one podium, and one or more tubercles for articulation of spines. The organization of ambulacral ossicles in cidaroid and Paleozoic echinoids is simple, but those of almost all extant echinoids are grouped together into compound or pseudocompound plates (Smith 1984). Each compound plate is a tagma with multiple pore pairs and usually one enlarged tubercle that bears a large spine (Fig. 8B); smaller tubercles of the plate support smaller spines. In pseudocompound plates of echinothurioid and holectypoid echinoids, one ossicle bears the enlarged tubercle; in compound plates, the tubercle is shared among two or more ambulacral ossicles. Trigeminate compound plates consist of three ossicles and their three pore pairs (Fig. 8B), and they are typical of the radially symmetrical (“regular”) sea urchins; but the plates of some are polyporous or polygeminate with 4–15 ambulacral ossicles (Smith 1988). Furthermore, not all ossicles in a compound plate might extend from the midline suture of the ambulacrum to the suture with an adjacent interambulacral plate. The organization of each tagma, therefore, varies widely among echinoid families (Smith 1984).

**Fig. 9:**
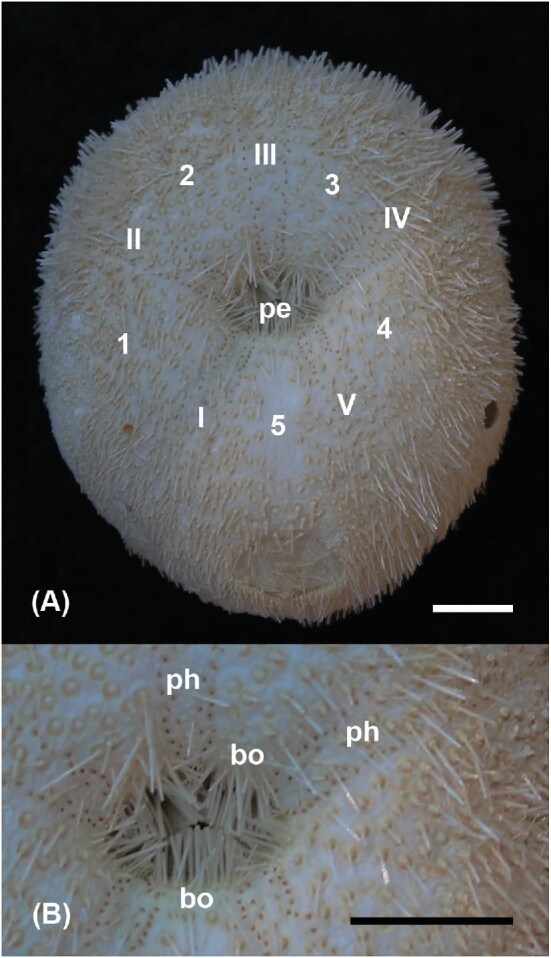
Bourrelets and phyllodes of *Echinolampas depressa*. (A) Whole specimen, oral view with the peristome (pe) at adoral junction of the ambulacral (pore-bearing) and interambulacral columns. Columns are labeled according to the Lovén System: ambulacrals in Roman numerals, interambulacrals in Arabic numerals. Scale bar 5 mm. (B) Enlarged view of peristome and surrounding regions of ambulacral and interambulacral columns. Bourrelets (bo) are clusters of specialized spines borne on the basal interambulacral ossicles, labeled here in interambulacra 3 and 5. Phyllodes (ph) are groups of basal ambulacral ossicles with enlarged podial pores, labeled here in ambulacra III and IV. Scale bar 5 mm.

In addition to the compounding of ambulacral ossicles, phyllodes and bourrelets (Fig. 9) are tagmata near the peristome (oral region; Hyman 1955; Kier 1962; Smith 1984). Phyllodes are groups of specialized ambulacral ossicles with either large pores for muscular podia to attach to substrata in high-energy habitats or dense clusters of small pores for small podia to collect food in small-grained substrata (Barras 2008). Bourrelets are clusters of swollen first interambulacral ossicles with numerous tubercles that bear small circumoral spines that manipulate food particles (Kier 1962; Gladfelter 1978).

**Fig. 10:**
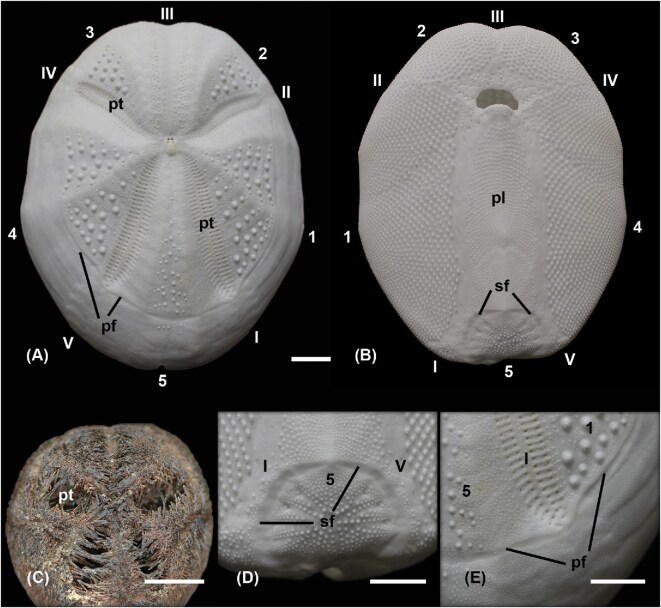
Tagmata of heart urchins. (A) Aboral and (B) oral views of *Plagiobrissus grandis* with petals (pt), plastron (pl), peripetalous fasciole (pf) and subanal fasciole (sf). Ambulacrum III is not petaloid. (C) *Abatus cordatus* with four petals (pt) sunken as brood chambers. (D) *Plagiobrissus grandis*, enlarged view of subanal fasciole (sf) in (B) crossing interambulacrum 5 and ambulacra I and V; compare with Fig. 11B. (E) *Plagiobrissus grandis*, enlarged view of peripetalous fasciole (pf) in (A) crossing ambulacrum I and interambulacra 1 and 5. All scale bars 1 cm.

**Fig. 11:**
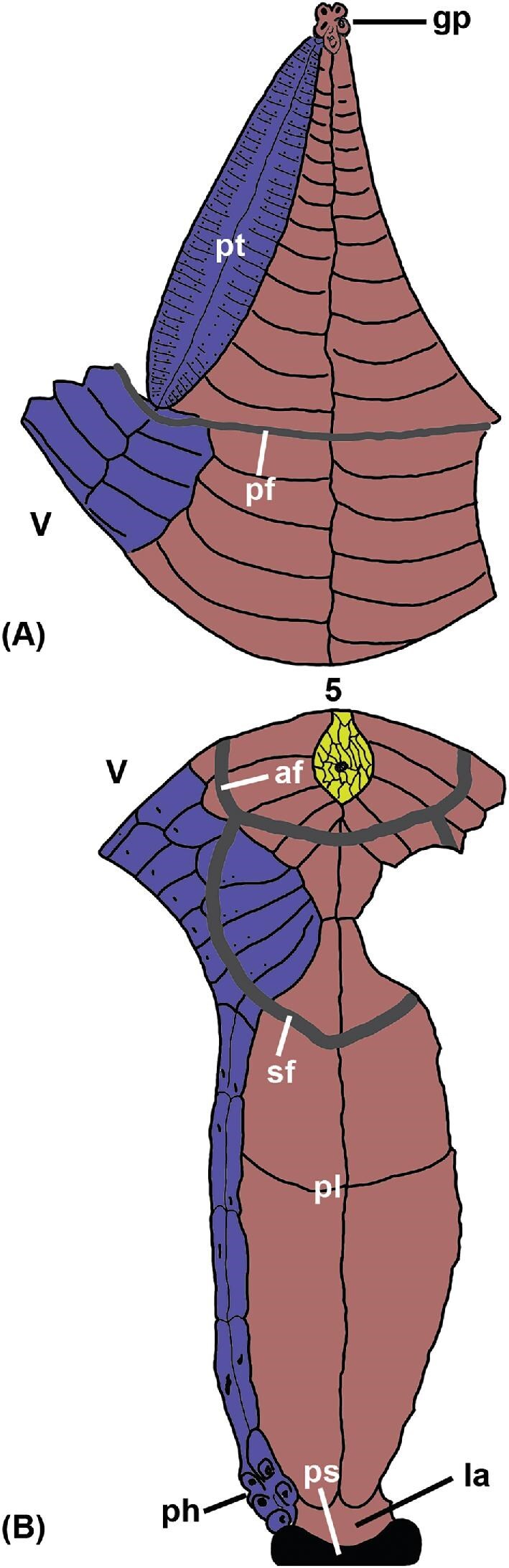
Tagmata of *Plagiobrissus grandis*, redrawn from Hyman (1955). Aboral (A) and oral (B) views of ambulacrum V (blue) and interambulacrum 5 (red). A few members of each column at the edge of the test are out of view. (A) A cluster of genital plates (gp) is at the apex of the interambulacral columns. The peripetalous fasciole (pf, gray) crosses one pair of interambulacral plates and the pair of ambulacral plates just proximal to the petal (pt). (B) From the peristome (ps), a group of four large interambulacral plates forms a plastron (pl) between the labrum (la) and the perianal region. The plastron is flanked by several pairs of elongated and narrow ambulacal plates that extend from the phyllode (ph) to a series of short and wide ambulacral plates in the perianal region. The subanal fasciole (sf) crosses the more distal pair of plastron plates and a cluster of wide ambulacral plates. The anal fasciole (af) encompasses the periproct (yellow) and crosses the wide interambulacral plates in the perianal region.

The bilaterally symmetrical tests of irregular urchins (Fig. 10; sand dollars, heart urchins, and relatives) are usually strongly tagmatized along the ambulacral and interambulacral columns (Fig. 11; Hyman 1955; Smith 1984). The ambulacra might have phyllodes proximally, followed by unspecialized ambulacral plates on the oral surface (Fig. 11B). On the aboral surface, ambulacral plates are formed into petaloid clusters with respiratory podia (Figs. 10A, 11A). The petals of brooding species often are sunken into the test as brood chambers in females (Fig. 10C; Hyman 1955; Schinner and McClintock 1993). The interambulacra might have proximal bourrelets, and the posterior interambulacrum (interambulacrum 5 in the Lovén System) typically is broadened as a plastron on the oral surface (Fig. 11B). The posterior interambulacrum also bears the anus in many families. Finally, the series of ambulacral and interambulacral plates might be crossed by bands (fascioles; Figs. 10D, E and 11) of dense, small, spine-bearing tubercles with highly ciliated spines that generate water currents (Smith and Stockley 2005).

### Holothuroidea

Extant cucumbers do not express metamerism (Turner 2024). The axial components of the adult body are restricted to the anterior (oral) end and comprise the ring canal, calcareous ring (in most holothuroids), and tentacles, five of the latter derived from the primary podia (Mooi and David 1997). The “longitudinal water vessels” (Mooi and David 1997) are interradial and do not originate from the primary podia; those species that bear podia (most Actinopoda; Miller et al. 2017) do not produce them according to the OPR (Mooi and David 1997). Extant holothuroids among the Echinodermata are, therefore, analogous to sipunculans and echiurans of the Annelida with reduced or absent metamerism and tagmosis (Struck et al. 2007).

## Discussion

Clark (1964) wrote extensively about the role of metamerism in locomotion of arthropods and annelids, including how metamerism, septation of the coelom, and the loss of septation work together with appendages or a hydrostatic skeleton to accomplish movement, either on surfaces or in soft sediments. Others (Russell-Hunter 1979; Willmer 1990; Rouse and Pleijel 2001; Hughes 2003; Fusco and Minelli 2013; Dunlop and Lamsdell 2017; Osawa et al. 2023) additionally addressed the occurrence of tagmosis in metameric groups; tagmosis can permit a change in the style of movement or provide body regions that serve other functions (e.g., gas exchange, defense, reproduction, generation of water currents).

Among the annelids, the clitellate earthworms and leeches (Oligochaeta, Hirudinea) show minimal tagmosis (Brusca et al. 2023). They have a head tagma of the prostomium and peristomium (some with additional anterior segments) and the clitellum; the clitellum is, however, an epidermal, not mesodermal, feature. In leeches, several segments form the anterior and posterior suckers. Some aquatic oligochaetes have tagmata with gills. Aside from a head tagma, polychaetous annelids demonstrate little tagmosis (“regionation”; Rouse and Pleijel 2001), most strongly developed in subclass Sedentaria. Russell-Hunter (1979) gave examples from this subclass: *Arenicola* (a burrower) with four tagmata and *Chaetopterus* (a tube dweller) with seven tagmata.

Arthropods strongly demonstrate tagmosis (Brusca et al. 2023), and the patterns of tagmosis expressed among the arthropods are highly diverse (Fusco and Minelli 2013). The basic tagmata are the head, thorax, and abdomen, although other terms are used in different groups; the terms are not always homologous. The appearance of tagmosis is strengthened by specialization of appendages and fusion of segments; and it is lessened by the joining of two of the three basic tagmata into a cephalothorax or a trunk. Millipedes provide an interesting case of tagmosis with the fusion of adjacent segments into pairs as diplosegments during embryogenesis by a mismatch between dorsal tergites and ventral sternites; their relatives, the pauropods and symphylans, have other patterns of segmental mismatches leading to numerous serial tagmata (Janssen 2011).

Tagmosis is also strongly developed in the Vertebrata in association with the axial skeleton, central and peripheral nervous systems, and skeletal musculature (Kardong 2019). Although not metamerically organized, endodermal organs become regionalized in concert with the tagmata. The tagmata might simply be the head, trunk, and tail, or further regionation can occur in the trunk.

Almost all extant echinoderms express some level of tagmosis along their metameric axes. The exception is the sea cucumbers, the vermiform extraxial body of which works like an aseptate hydrostat (Clark 1964). Proximal components of the axial skeleton form a perioral tagma that frames the mouth. Somewhat more distal segments might strengthen the disc or calyx, especially in groups with branching axes. Sections of the axes free of the central body can be tagmatized further for protection, reproduction, and feeding. In the special case of echinoids, with no free axes, segments of the ambulacral series in most groups are tagmatized into compound plates that strengthen the test by effectively reducing the number of components without reducing the number or density of podia. Both ambulacral and interambulacral columns in the irregular echinoids often have tagmata for burrowing, respiration, broodcare, and feeding. Conformance of body plans to the EAT (Mooi et al. 1994; David et al. 2000) has been evaluated for all of the numerous fossil classes of Echinodermata, and some of those several groups considered here express tagmosis distal to the perioral tagma, especially among taxa with branched ambulacra.

Pease (1990) wrote a strongly critical review of Willmer’s (1990) book for her rejection of cladistics and her reliance on “adaptational ‘story telling’ arguments.” Among the many statements that troubled Pease might have been Willmer’s that “tagmosis... is a predictable consequence of segmentation.” Despite its arguable value in Willmer’s “pragmatic approach” to patterns of animal evolution, that statement at least prompted the present essay. It is interesting that there is a fourth group of animals that have evolved various degrees and patterns of tagmosis along their metameric axes.

## Data Availability

Not applicable.
